# Head and neck myxofibrosarcoma: a case report and review of the literature

**DOI:** 10.1186/1752-1947-8-468

**Published:** 2014-12-29

**Authors:** Giovanni Dell’Aversana Orabona, Giorgio Iaconetta, Vincenzo Abbate, Pasquale Piombino, Antonio Romano, Fabio Maglitto, Giovanni Salzano, Luigi Califano

**Affiliations:** 4 Department of Maxillofacial surgery, University of Naples “Federico II”, Naples, Italy; 5 Department of Neurosurgery, University of Salerno, Salerno, Italy; 6 MaxilloFacial Surgery Unit, Department of Neurosciences and Reproductive and Odontostomatological Sciences, Federico II University of Naples, Via Pansini, 5, 80131 Naples, Italy

**Keywords:** Malignant fibrous histiocytoma, Midcheek masses, Myxofibrosarcoma, Myxosarcoma

## Abstract

**Introduction:**

Myxofibrosarcoma is the most common soft tissue sarcoma that occurs in late adult life, peaking in the seventh decade, and it is mainly encountered in the lower extremities. Myxofibrosarcoma of the head and neck are extremely rare. To the best of our knowledge, only 19 cases have been described in the head and neck so far. This is a literature review and retrospective chart review of our experience in head and neck myxofibrosarcoma treatment in our department.

**Case presentation:**

In this case report we describe a 35-year-old Caucasian man who presented the first case of myxofibrosarcoma arising from the pterygopalatine fossa. The peculiar anatomical location and the extent in the midcheek region make this case a hard “challenge” for the surgeon, in order to guarantee wide surgical margins of resection. A total right maxillectomy was accomplished by means of the Weber-Ferguson approach, preserving the orbital floor. The excised portion was reconstructed using the free rectus abdominis myocutaneous flap. Postoperative radiotherapy was given to the area adjacent to the lesion, with a total dose of 60Gy. No relapse occurred in the 27-month postoperative follow-up.

**Conclusions:**

The case described suggests the importance of combined surgical and adjuvant radiotherapy to avoid local and distant recurrences of the tumor. In our opinion, combined surgical and adjuvant radiotherapy followed by close clinical observation to search for a metastatic disease is advisable in all cases. Further studies are needed to confirm the efficacy of combined radio-chemotherapy for head and neck myxofibrosarcoma in terms of long-term disease-free survival.

## Introduction

The terms “myxofibrosarcoma” (MFS) and “myxosarcoma” refer to a connective tissue neoplasm of fibroblastic origin set in myxoid matrix which has been classified by O’Brien and Stout [1] as a myxoid variant of malignant fibrous histiocytoma (MFH) [2, 3]. In 2002, the World Health Organization [4] declassified the MFH as a diagnostic entity and determined that myxoid MFH without myogenic, lipoblastic and chondrogenic features be diagnosed as MFS. MFS is the most common soft tissue sarcoma that occurs in late adult life, peaking in the seventh decade and is mainly encountered in the lower extremities (77%), trunk (12%), retroperitoneum or mediastinum (8%). Only a few corresponding cases in the head and neck region have been reported to date, with 3 to 10% involvement. This is a literature review and retrospective chart review of our experience in head and neck MFS treatment in our department. The purpose of this study is to provide the reader with an overview of the pathology of this complex anatomic area focusing attention on the differential diagnosis and recent treatment strategies. Furthermore we describe the first case of MFS arising from the pterygopalatine fossa extending to the soft tissues of the midcheek area. We discuss the clinical, histological, immunohistochemical and ultrastructural features of these large neoplasms in such a rare and critical anatomical area.

## Case presentation

**Figure 1:**
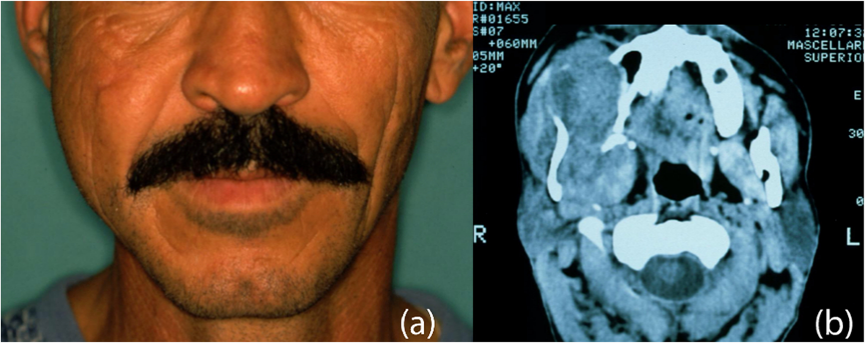
**Preoperative patient’s study. (a)** Patient’s facial appearance. **(b)** Axial computed tomography scan showing the extent of the tumor.

**Figure 2:**
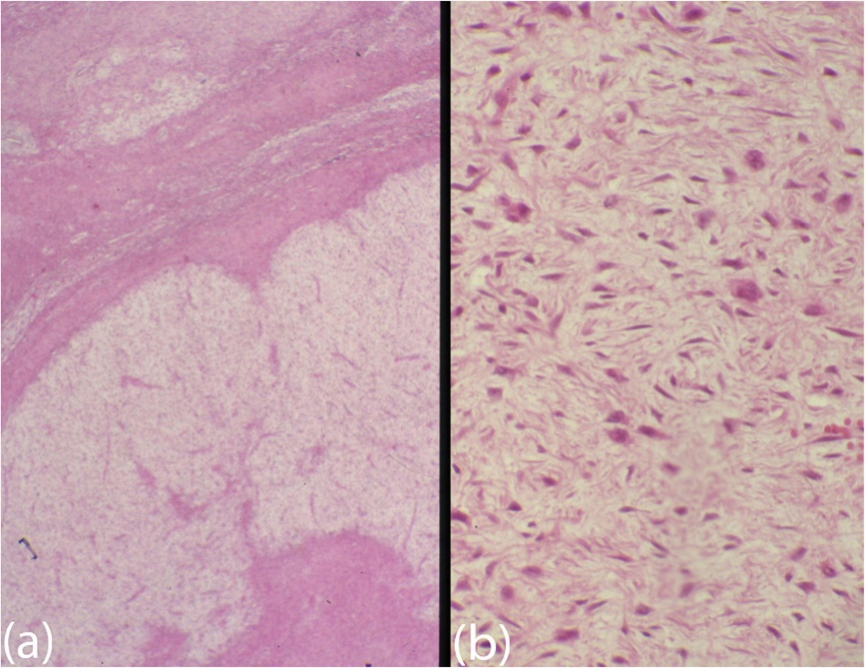
**Histological appearance. (a)** Photomicrograph showing myxoid nodules and fibrous septa. Hematoxylin and eosin original magnification ×40. **(b)** Photomicrograph showing spindle-shaped cells in a fascicular arrangement. Hematoxylin and eosin, original magnification ×100.

A 35-year-old Caucasian man presented with a complaint of facial asymmetry for a mass in his right midcheek area, which had increased in size over 4 months (Figure 1a). In January 2011, he was admitted to our Department of Maxillofacial Surgery complaining of right cheek discomfort and pain, refractory to analgesics, in his right maxilla area and radiating to the right zygoma. Two weeks before the onset of pain, he underwent an examination by his dentist. When he came to our observation, an apparent swelling of the right side of his face was observed, with anesthesia, in the area of his face innervated by the second branch of the trigeminal nerve, and he had pain on palpation. An intraoral examination revealed an expansion of his upper right gums and the vestibular portion, and the mucosa appeared reddened, and his teeth were movable with the presence of a widespread periodontitis. No lymphadenopathy was apparent at neck examination. Computed tomography revealed a hyperdense mass lesion, measuring 83 × 55mm in its greatest dimension, arising from the pterygopalatine fossa, and extending to the soft tissue of his midcheek area (Figure 1b). An open biopsy was conducted for the diagnosis. The biopsy specimen was histologically examined; it was found to be composed of fibroma and exhibited myxomatous changes as well as abundant collagen fibers. The tumor was characterized by spindle-cell proliferation with moderate cellular density in the fibromyxoid stroma. On immunohistochemical examination, the tumor cells were positive for vimentin, Ki-67, smooth muscle actin, but negative for S-100 (Figure 2a,b).

**Figure 3:**
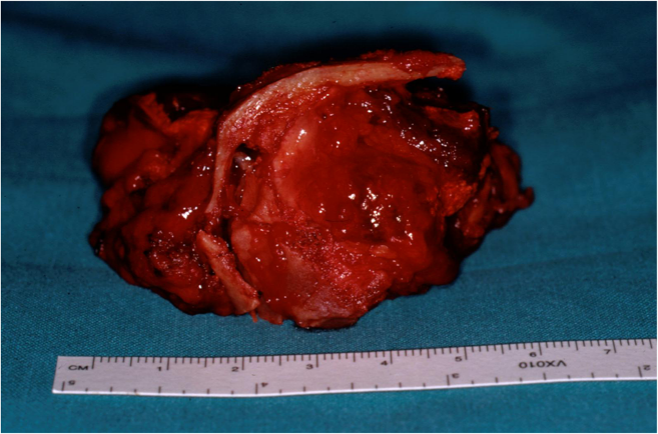
**Overall view of the tumor.**

A total right maxillectomy was accomplished by means of Weber-Ferguson approach, preserving the orbital floor. On macroscopic examination the anterior and the posterior maxillary walls were totally reabsorbed and the tumor was removed from the pterygopalatine fossa (Figure 3). In the right midcheek area, the tumor was detached from the subcutaneous tissues with safe margins preserving the skin. The excised portion was reconstructed using the free rectus abdominis myocutaneous flap. The size of the excised tumor was approximately 84 × 56 × 58mm; it was well circumscribed, elastic, and homogeneously whitish in color. The final diagnosis was low-grade MFS. Postoperative radiotherapy was given to the area adjacent to the lesion, with a total dose of 60Gy. No relapse occurred in 27-month postoperative follow-up.

## Discussion

MFS of the head and of the neck are extremely rare. To the best of our knowledge, only 19 cases have been previously described in the head and neck so far (Table 1), our case being the first observed within the pterygopalatine fossa. Apart from the general histologic features, MFS demonstrates wide histologic variability, based on its grade of malignancy. These tumors have been subdivided into three or four grades based on the degree of cellularity, nuclear pleomorphism, and mitotic activity [5, 6]. Low-grade MFSs are considered to have low malignancy, and rarely show distant metastasis, implying a good short-term prognosis. Mentzel *et al.* [5], in their study, reviewed 75 patients and documented local recurrence in 55% of cases and distant metastases in 33%. More recently, Nishio *et al.* [7] reported an overall 5-year survival rate of 60 to 70%; however, the local recurrence rate of the low-grade type is as high (50% to 60%) as that of the high-grade type. Sanfilippo *et al*. [8], in a review of 178 patients, stated that, while local recurrences were predicted by the quality of surgical margins, the distant metastases were observed based on histological grade. Zhu Qiubei *et al.* [9] reviewed, from 1981 until 2012, 18 cases. The average age of onset reported was 53 years with male prevalence (66.7%). The average follow-up after treatment for all the cases of MSF of head and neck described was 17 months (range 3 to 39 months). None of the patients reported developed locoregional lymph node or systemic metastases at the latest follow-up. Surgery followed by adjuvant radiotherapy was the most common form of treatment strategy, and only two articles dealing with radio/chemotherapy exist, due to the small number of cases. Cante *et al.* [10] reported one case of MSF of maxillary sinus with a complete remission at 18-month follow-up after a combined radio-chemotherapy (RT/CHT) without surgery (Table 2). The results obtained with combined RT/CHT treatment for head and neck MFS should encourage further studies to confirm the efficacy in terms of long-term disease-free survival. According to these considerations, a complete tumor resection with adequate resection margins, followed by adjuvant radiotherapy, remains the mainstay for treatment of MFS. A possible re-excision of recurrent lesions is considered the mainstay of therapy for disease control.

**Table 1:** **Cases of myxofibrosarcoma in the head and neck region**

Region	Cases
Maxillary sinus	3
Sphenoid sinus	3
Orbit	2
Maxilla	2
Esophagus	1
Hypopharynx	1
Neck	1
Vocal folds	1
Parotid	3
Mandible	1
Infratemporal space	1
**Total of cases reported in head and neck**	**19**

**Table 2:** **Head and neck myxofibrosarcoma: review of cases**

Sex	Age (years)	Treatment	Follow-up
Male	66	Radiotherapy	3 months
Male	58	Surgery	Unknown
Female	67	Surgery, radiotherapy	8 months
Female	52	Surgery	Unknown
Male	55	Surgery	8 months
Male	Unknown	Unknown	Unknown
Male	40	Surgery	Unknown
Male	69	Surgery	16 months
Male	55	Surgery	27 months
Female	36	Surgery	24 months
Male	79	Surgery	Unknown
Female	37	Surgery, radiotherapy	8 months
Female	27	Surgery, radiotherapy	6 months
Male	23	Surgery, radiotherapy	39 months
Male	69	Radiotherapy, chemotherapy	12 months
Female	78	Surgery, radiotherapy	24 months
Female	42	Surgery, radiotherapy	26 months
Male	52	Surgery, radiotherapy	20 months
Male	65	Radiotherapy, chemotherapy	18 months

## Conclusions

In conclusion, the peculiar anatomical location and the extent in midcheek region, make such a case a hard “challenge” for the surgeon in order to guarantee wide surgical margins of resection. Combined surgical and adjuvant radiotherapy, in order to avoid local and distant recurrences of the tumor, is absolutely recommended. An aggressive follow-up in order to search for a metastatic disease is advisable in all cases. Further studies are needed to confirm the efficacy of combined RT/CHT for head and neck MSF in terms of long-term disease-free survival.

## Consent

Written informed consent was obtained from the patient for publication of this case report and accompanying images. A copy of the written consent is available for review by the Editor-in-Chief of this journal.

## Abbreviations

MFH: Malignant fibrous histiocytoma
MFS: Myxofibrosarcoma
RT/CHT: Combined radio-chemotherapy.
